# High Accuracy Acquisition of 3-D Flight Trajectory of Individual Insect Based on Phase Measurement

**DOI:** 10.3390/s16122166

**Published:** 2016-12-17

**Authors:** Cheng Hu, Yunkai Deng, Rui Wang, Changjiang Liu, Teng Long

**Affiliations:** Beijing Key Laboratory of Embedded Real-Time Information Processing Technology, School of Information and Electronics, Beijing Institute of Technology, Beijing 10081, China; cchchb@163.com (C.H.); 3120140313@bit.edu.cn (Y.D.); liuchangjiang@bit.edu.cn (C.L.); longteng@bit.edu.cn (T.L.)

**Keywords:** insect migration, individual insect, 3-D flight trajectory, range measurement, angle measurement

## Abstract

Accurate acquisition of 3-D flight trajectory of individual insect could be of benefit to the research of insect migration behaviors and the development of migratory entomology. This paper proposes a novel method to acquire 3-D flight trajectory of individual insect. First, based on the high range resolution synthesizing and the Doppler coherent processing, insects can be detected effectively, and the range resolution and velocity resolution are combined together to discriminate insects. Then, high accuracy range measurement with the carrier phase is proposed. The range measurement accuracy can reach millimeter level and benefits the acquisition of 3-D trajectory information significantly. Finally, based on the multi-baselines interferometry theory, the azimuth and elevation angles can be obtained with high accuracy. Simulation results prove that the retrieval accuracy of a simulated target’s 3-D coordinates can reach centimeter level. Experiments utilizing S-band radar in an anechoic chamber were taken and results showed that the insects’ flight behaviors and 3-D coordinates’ variation matched the practical cases well. In conclusion, both the simulated and experimental datasets validate the feasibility of the proposed method, which could be a novel measurement way of monitoring flight trajectory of aerial free-fly insects.

## 1. Introduction

Insects have evolved two general strategies to cope with habitat changes: diapause or migration. Long-distance migration, a seasonal to and from movement of insect populations between different regions where conditions are alternately favorable or unfavorable, is a widespread phenomenon among animals [1]. Billions of insects migrate annually, which makes it an important reason for crop pests’ sudden outbreaks and plant disease prevalence [2]. Therefore, better understanding of insect migration could help us develop more effective management strategies against major crop pests and other relevant diseases.

Insects, together with the fact that most species are nocturnal flying hundreds of meters above the ground, are too small for movement observing and individual tracking. Therefore, knowledge of insect migration lags behind that of other vertebrates, such as birds and bats. However, radar provides a good solution to this difficult problem, with the remote sensing capabilities of extracting information of free-fly migratory insects [3,4]. With these direct parameters, new discoveries of ecological and biological behaviors of migration insects were published constantly. For example, the phenomena of large-scale taking-off, high-density concentrating, and downwind orientation of insect swarms were directly observed by entomological radars [5,6,7,8].

Although important progresses have been made by entomological radar studies, the accurate acquisition of 3-D flight trajectory of individual insect remains unsolved by the limitation of working modes and system functions of traditional entomological radars. Nowadays, entomological radars can be mainly divided into two types: scanning radar and vertical-looking radar (VLR). Scanning radar can acquire migration parameters for the overflying population, such as density, speed, and flight direction. However, it is difficult for scanning radar to acquire information of individual insect [9]. VLR allows information related to the size, shape, wingbeat frequency and body alignment of overflying insects to be acquired. However, the narrow beam it emits determines that the spatial scale it monitors is quite small. Besides, the range information is measured with the time delay of the pulse signal and angle information is deduced from the echo intensity modulation. Therefore, VLR can just acquire the flight trajectory of individual insect at a very small spatial scale with low accuracy [10].

Acquisition of the flight trajectory of migratory insects can improve our knowledge on the migration behavior of insects, and help us model accurately the migration pathway of crop pests. For example, with high accuracy acquisition of the insects’ flight trajectories, it is possible to figure out how different insects adjust their flight to orient towards the same direction [11]. As another example, entomologists found that concentrating behavior related with rainfall could cause large numbers of insects to land within a short time and pest outbreaks would happen [12]. Combining trajectory information with rainfall information, effective early warning of pest outbreaks could possibly be realized. Therefore, studies on new methods of obtaining 3-D flight trajectory of migratory insects are imperative and important.

The 3-D location of a target can be determined by its range, elevation angle and azimuth angle in the polar coordinate. The direction of arrival (DOA) of an incoming signal can be determined with a multi-baselines interferometer. Although the elevation and azimuth angles of a target can be determined with high accuracy, the DOA method has a disadvantage of strict requirement of baseline settings to eliminate influence of phase ambiguity [13]. The time summation of arrival (TSOA) method can also be used to determine a target’s 3-D location. However, this method needs to solve non-linear equations with iteration calculation. The computationally time-consuming process means it cannot be used to acquire trajectory information of insects in real-time [14]. Till now, no research on acquiring the 3-D flight trajectory of migratory insects in entomological radar applications has been carried out.

To address the above problem, a novel method to acquire the 3-D flight trajectory of individual insect is proposed. First, based on the high range resolution synthesizing and the Doppler coherent processing, insects can be detected effectively, and the range resolution and velocity resolution are combined together to discriminate insects. Then, high accuracy range measurement with the carrier phase is proposed. The range measurement accuracy can reach millimeter level and benefits the acquisition of trajectory information significantly. Finally, based on the multi-baselines interferometry theory, the azimuth and elevation angles can be obtained with high accuracy. We utilize S-band (wavelength 9.09 cm) radar, which adopts high resolution technique and employs multi-baselines interferometry theory, to carry out experiments of several typical migratory insects in an anechoic chamber. Both the simulated and experimental datasets validate the feasibility of the proposed method, which lays theoretic foundation for monitoring the 3-D flight trajectories of aerial free-fly insects.

The remainder of the paper is organized as follows: Section 2 introduces the proposed method, which includes five steps, i.e., signal model, high range resolution synthesizing, Doppler coherent processing, high accuracy range measurement with carrier phase, and angle measurement with multi-baselines interferometry. Section 3 demonstrates the performance of the proposed method with the simulated and experimental datasets. Our conclusions are drawn in Section 4.

## 2. Method

### 2.1. Signal Model

Water is a highly effective scatter of radio waves. An insect’s body contains a mass of water, which makes it an identifiable target of radar. However, in contrast to the range resolution (decimeter or meter level) of current radars systems, insects are too small (millimeter or centimeter level). Therefore, an insect can be regarded as a point target.

**Figure 1:**
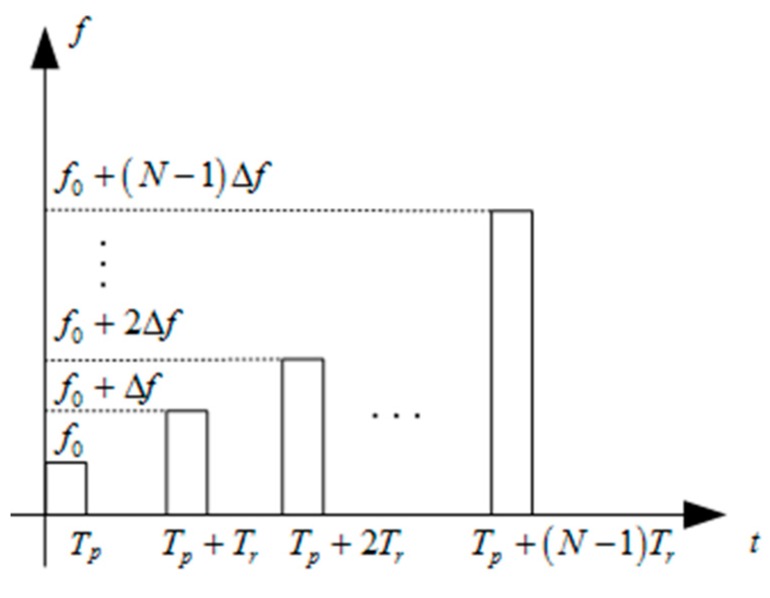
Scheme of the stepped-frequency signal.

The stepped-frequency technique is adopted to realize high range resolution [15]. A frame of stepped-frequency signal includes a group of subpulses. The difference of the carrier frequencies between two neighboring subpulses is the same, and it is called the frequency step. Figure 1 shows the scheme of the stepped-frequency signal. The transmitted signal can be modeled by

(1) $$S(t)=\sum_{i=0}^{N-1}\mathrm{rect}\left(\frac{t-iT_{r}-T_{p}/2}{T_{p}}\right)\cdot \exp\left[j2\pi \left(f_{0}+i\Delta f\right)t\right],$$

where $T_{p}$ is the subpulse duration, $T_{r}$ is the subpulse repetition interval, Δf is the frequency step size, *N* is the number of subpulses in a frame of stepped-frequency signal and $f_{0}$ is the carrier frequency of the first subpulse. rect[⋅⋅⋅] is a rectangular window function which limits the time range $iT_{r}\leq t\leq iT_{r}+T_{p}$. Every subpulse is a linear frequency-modulated signal.

In ideal conditions, as a point target, echo amplitude of individual insect can be considered as one. Therefore, for a moving insect with initial distance $R_{0}$ and radial velocity *v*, the time delay between its echo signal and transmitted signal is τ(t)=2(R−vt)/c, where *c* is the speed of light. The echo signal can be described by

(2) $$S_{r}(t)=\sum_{i=0}^{N-1}\mathrm{rect}\left(\frac{t-iT_{r}-T_{p}/2-\tau \left(t\right)}{T_{p}}\right)\exp\left[j2\pi \left(f_{0}+i\Delta f\right)\left(t-\tau \left(t\right)\right)\right].$$

Considering the coherent referenced signal as

(3) $$S_{p}(t)=\sum_{i=0}^{N-1}\mathrm{rect}\left(\frac{t-iT_{r}-T_{r}/2}{T_{r}}\right)\exp\left[-2\pi \left(f_{0}+i\Delta f\right)t\right].$$

Mixing the echo signal with the referenced signal, we have

(4) $$U(t)=S_{r}\left(t\right)S_{p}^{*}\left(t\right)=\sum_{i=0}^{N-1}\mathrm{rect}\left(\frac{t-iT_{r}-T_{p}/2-\tau \left(t\right)}{T_{p}}\right)\exp\left(-j2\pi f_{0}\tau \left(t\right)\right)\exp\left(-j2\pi i\Delta f\tau \left(t\right)\right).$$

### 2.2. High Range Resolution Synthesizing

In order to acquire the flight trajectory of individual insect, both ability to acquire range information with high accuracy and ability to distinguish individual insect from insects group are necessary. Traditional entomological radars emit pulse signal and the typical values of the pulse durations are 0.1, 0.07 or even 0.05 μs (15, 10 and 7.5 m range resolution, respectively). The low range resolution makes it easy to have multiple insects in a resolution cell. Therefore, we adopt high resolution synthesizing technique [16,17].

The total time $T_{F}=N\cdot T_{r}$ of each frame of the stepped-frequency signal is in the order of millisecond and the radial flight velocity of an insect is no more than 10 m/s, then the flight distance of individual insect in $T_{F}$ is very short, which means the time delay τ(t) can be considered as a constant τ. The first term of Equation (4) limits the time range of the echo signal, the second term $\exp(-j2\pi f_{0}\tau )$ is a constant, while the third term exp(−j2πiΔfτ) can be regarded as a frequency-domain signal with linear change frequency at τ. Sample the mixing result at time $t=iT_{r}+\tau$ and get

(5) $$U{|}_{t=iT_{r}+\tau }=\exp\left(-j2\pi f_{0}\tau \right)\exp\left(-j2\pi i\Delta f\tau \right).$$

Take the IDFT (inverse discrete Fourier transform) operation of Equation (5),

(6) $$\begin{array}{ll} V\left(n\right) & =\frac{1}{N}\sum_{i=0}^{N-1}\exp\left(-j2\pi f_{0}\tau \right)\exp\left(-j2\pi i\Delta f\tau \right)\cdot \exp\left(j\frac{2\pi i}{N}n\right)\\ & =\frac{1}{N}\exp\left(-j2\pi \left(f_{0}+\frac{\left(N-1\right)\Delta f}{2}\right)\tau \right)\cdot \exp\left(j\pi \left(1-\frac{1}{N}\right)n\right)\cdot \frac{\sin\left(\pi \left(n-\frac{R}{\Delta R}\right)\right)}{\sin\left(\frac{\pi }{N}\left(n-\frac{R}{\Delta R}\right)\right)} \end{array}.$$

where n=0,1,…,N−1, and ΔR=c/(2N⋅Δf) is the range resolution. V(n) is the high resolution synthesizing result and a frame of wideband signal in time domain. Its amplitude is

(7) $$\left|V\left(n\right)\right|=\left|\frac{\sin\left(\pi \left(n-\frac{R}{\Delta R}\right)\right)}{\sin\left(\frac{\pi }{N}\left(n-\frac{R}{\Delta R}\right)\right)}\right|.$$

|V(n)| is a discrete *sinc* function. When *n* equals R/ΔR, |V(n)| gets its max value. The range resolution ΔR commonly used in the high resolution radar systems is in decimeter level. Considering that *n* is an integer and R/ΔR might not be, range measurement error cannot be avoided if the target range *R* is just determined by *n*, which is the location of the max amplitude of the high resolution synthesizing result. The measurement error is approximately several centimeters or decimeters.

Adoption of the high resolution synthesizing technique benefits the distinction of individual insect from insects group. Besides, high resolution synthesizing can improve signal-to-noise ratio (SNR), since the synthesized result is a *sinc* function and the signal power is concentrated. The detection performance of weak targets can be improved greatly with the high resolution technique.

### 2.3. Doppler Coherent Processing

Current research has proven RCS (Radar Cross Section) of individual insect is very small and RCS of tiny insects can even lower to −80 dBsm [18,19]. Therefore, random noise affects the effective detection of individual insect. The phase variation of the random noise is stochastic, while that of the high resolution synthesizing result is coherent. Doppler processing is a coherent integration method, so it can be used to further improve SNR and benefits the detection of insects flying at the high altitude [20].

To acquire 3-D flight trajectory of individual insect on a large spatial scale, a wide beam is essential. However, it causes a large resolution cell and makes it easy for multiple insects to fly in the same resolution cell. Doppler processing can acquire velocity information based on the shift of Doppler frequency and provide another dimension of velocity resolution. Therefore, multiples insects flying in the same resolution cell can be discriminated in a new 2-D joint domain of range domain and velocity domain.

The Doppler processing method takes DFT (discrete Fourier transform) operation to multi-frames of high resolution synthesizing results. Assume that multi-frames of high resolution synthesizing results constitute a group and each group includes *M* frames. If too many frames are used to take Doppler processing, the flying distance of an insect within the time of *M* frames may cross multiple range resolution cells and then velocity compensation is essential to make effective Doppler analysis. To simplify this problem and avoid velocity compensation, *M* should not be too large. Assuming the insect target moves uniformly relative to the radar in each group, the time delay between the echo and transmitted signals is

(8) $$\tau \left(t\right)=\frac{2\left(R_{0}-vt\right)}{c}=\frac{2\left(R_{0}-mT_{F}\cdot v\right)}{c}\;m=0,1,\ldots ,M-1,$$

where $R_{0}$ means the initial distance between the insect target and the radar. The high resolution synthesizing result in Equation (6) can be further described as

(9) $$\begin{array}{lll} V\left(n,m\right) & = & \exp\left(-j2\pi \left(f_{0}+\frac{\left(N-1\right)\Delta f}{2}\right)\left(\frac{2\left(R_{0}-mT_{F}v\right)}{c}\right)\right)\\ & & \;\cdot \exp\left(j\pi \left(1-\frac{1}{N}\right)n\right)\cdot \frac{1}{N}\frac{\sin\left(\pi \left(n-\frac{R_{0}-mT_{F}v}{\Delta R}\right)\right)}{\sin\left(\frac{\pi }{N}\left(n-\frac{R_{0}-mT_{F}v}{\Delta R}\right)\right)}\\ & = & \exp\left(-j2\pi \left(\frac{2\left(R_{0}-mT_{F}v\right)}{\lambda }\right)\right)\\ & & \;\cdot \exp\left(j\pi \left(1-\frac{1}{N}\right)n\right)\cdot \frac{1}{N}\frac{\sin\left(\pi \left(n-\frac{R_{0}-mT_{F}v}{\Delta R}\right)\right)}{\sin\left(\frac{\pi }{N}\left(n-\frac{R_{0}-mT_{F}v}{\Delta R}\right)\right)} \end{array},$$

where $\lambda =c/(f_{0}+(N-1)\Delta f/2)$ is the wavelength of the radar signal. *n* is a variable, so the complex term exp(jπ(1−1/N)n) should be compensated before taking Doppler processing. Compensating the complex term including *n* of Equation (9), we can get

(10) $$V\left(n,m\right)=\exp\left(-j2\pi \left(\frac{2\left(R_{0}-mT_{F}v\right)}{\lambda }\right)\right)\cdot \frac{1}{N}\frac{\sin\left(\pi \left(n-\frac{R_{0}-mT_{F}v}{\Delta R}\right)\right)}{\sin\left(\frac{\pi }{N}\left(n-\frac{R_{0}-mT_{F}v}{\Delta R}\right)\right)}.$$

When taking Doppler processing, regard *n* as a constant and *m* a variable, so the phase of the first term shows linear variation with *m*. The second term is a *sinc* function. When *n* equals $(R_{0}-mT_{F}v)/\Delta R$, the second term gets its max value 1, but *m* is a variable when take Doppler processing, so its max value will change with *m*. In order to avoid velocity compensation, the max value of the second term should approach 1 when *m* varies. Under the condition that the amplitude fluctuation of the max value is no more than 5%, we can get the condition that *M* should satisfy is

(11) $$MT_{F}v\leq \Delta R/7.$$

Now the max value of the second term can be regarded as a constant, so when taking Doppler processing, we can just consider the first term of Equation (10). Get the first complex term,

(12) $$g\left(m\right)=\exp\left(-j2\pi \left(\frac{2\left(R_{0}-mT_{F}v\right)}{\lambda }\right)\right).$$

Take Doppler processing to g(m),

(13) $$\begin{array}{ll} G\left(k\right) & =\frac{1}{M}\sum_{m=0}^{M-1}\exp\left(-j2\pi \left(\frac{2\left(R_{0}-mT_{F}v\right)}{\lambda }\right)\right)\exp\left(-j\frac{2\pi k}{M}m\right)\\ & =\frac{1}{M}\exp\left(-j\frac{4\pi }{\lambda }\left(R_{0}-\frac{\left(M-1\right)T_{F}v}{2}\right)\right)\cdot \exp\left(-j\pi \left(1-\frac{1}{M}\right)k\right)\frac{\sin\left(\pi \left(k-\frac{v}{\Delta v}\right)\right)}{\sin\left(\frac{\pi }{M}\left(k-\frac{v}{\Delta v}\right)\right)} \end{array},$$

where k=0,1,…,M−1, $\Delta v=\lambda /(2MT_{F})$ is the velocity resolution. In order to extract carrier phase of the target, the complex phase term exp(−jπ(1−1/M)k), including *k*, should be compensated and we can get

(14) $$\begin{array}{l} V\left(n,k\right)=DFT\left[V\left(n,m\right)\right]=\frac{1}{NM}\exp\left(-j\frac{4\pi }{\lambda }R_{M}\right)\cdot \frac{\sin\left(\pi \left(n-\frac{R_{M}}{\Delta R}\right)\right)}{\sin\left(\frac{\pi }{N}\left(n-\frac{R_{M}}{\Delta R}\right)\right)}\frac{\sin\left(\pi \left(k-\frac{v}{\Delta v}\right)\right)}{\sin\left(\frac{\pi }{M}\left(k-\frac{v}{\Delta v}\right)\right)} \end{array},$$

where $R_{M}=R_{0}-(M-1)T_{F}v/2$ is the mean value of the distance between the insect target and radar within the time of *M* frames. V(n,k) is the Doppler coherent processing result. Other than range resolution ΔR, V(n,k) also has velocity resolution Δv. Therefore, the Doppler processing result can provide another dimension of velocity resolution, which benefits the discrimination of insects in a new 2-D joint domain of range domain and velocity domain. Amplitude of V(n,k) is

(15) $$\left|V\left(n,k\right)\right|=\left|\frac{\sin\left(\pi \left(n-\frac{R_{M}}{\Delta R}\right)\right)}{\sin\left(\frac{\pi }{N}\left(n-\frac{R_{M}}{\Delta R}\right)\right)}\right|\cdot \left|\frac{\sin\left(\pi \left(k-\frac{v}{\Delta v}\right)\right)}{\sin\left(\frac{\pi }{M}\left(k-\frac{v}{\Delta v}\right)\right)}\right|.$$

|V(n,k)| is a discrete 2-D*sinc* function. When *n* equals R/ΔR and *k* equals v/Δv, |V(n,k)| reaches its maximum value. The complex information of the maximum value is

(16) $$V{|}_{n=R/\Delta R,k=v/\Delta v}=\frac{1}{NM}\exp\left(-j\frac{4\pi }{\lambda }R_{M}\right).$$

We can see that taking Doppler coherent processing to the high resolution synthesizing results is equivalent to making secondary compression. Moreover, the Doppler processing result has velocity resolution besides range resolution. In conclusion, with Doppler coherent processing, SNR can be further improved to benefit the detection of insects and velocity resolution is introduced to benefit discrimination of insects.

### 2.4. High Accuracy Range Measurement with Carrier Phase

From Equation (15), the Doppler processing result is a discrete 2-D *sinc* function. According to the location of the maximum amplitude, the target’s range *R* and radial velocity *v* can be determined. Traditional high resolution radars utilize the time delay of the echo signal, i.e., according to the location of the maximum amplitude of the Doppler processing results, to take range measurement. Common accuracy of range measurement with the time delay is in the order of centimeter or decimeter. The measurement accuracy is not high enough. To acquire 3-D flight trajectory of individual insect with high accuracy, high accuracy range measurement is essential.

Considering the phase term shown in Equation (16), the carrier phase is very sensitive to the radial range variation of the target and it is potential for high accuracy range measurement. The half-wavelength motion in range could cause 2π phase change. If the range change is larger than λ/2 between two adjacent groups to take Doppler processing, the phase difference could be ambiguous. Thus, the frame number of signals to take Doppler processing should be limited and high pulse repetition frequency is essential. After satisfying the unambiguous condition, the accurate range can be deduced with the carrier phase difference. Moreover, the Doppler processing result has a high SNR, so the accuracy of range measurement based on carrier phase difference can be ensured [21,22]. The accurate range information is quite critical for the acquisition of 3-D trajectory information.

From Equation (16), in an ideal case, carrier phase of the target is

(17) $$\varphi \left(t_{p}\right)=-\frac{4\pi R\left(t_{p}\right)}{\lambda },$$

where p=0,1,2,… means the $p_{th}$ group to take Doppler processing, $t_{p}$ is the discrete sampling interval and equals $MT_{F}$. The carrier phase difference of two adjacent groups is

(18) $$\Delta \varphi_{p+1}=\varphi \left(t_{p+1}\right)-\varphi \left(t_{p}\right)=-\frac{4\pi \left(R\left(t_{p+1}\right)-R\left(t_{p}\right)\right)}{\lambda }.$$

It can be noted that the phase difference information includes range difference information. Under the condition that $R(t_{p})$ is known, $R(t_{p+1})$ can be acquired with high accuracy from the phase difference.

(19) $$R\left(t_{p+1}\right)=R\left(t_{p}\right)-\frac{\lambda }{4\pi }\Delta \varphi_{p+1}.$$

Utilizing Equation (19), range information at any moment can be acquired as

(20) $$R\left(t_{p}\right)=R\left(t_{0}\right)-\frac{\lambda }{4\pi }\sum_{l=1}^{p}\Delta \varphi_{l},$$

where $R_{0}$ is the initial range.

The accuracy of range measurement is determined by the extraction accuracy of carrier phase difference. Actually, insects’ RCS are related with their aspect—the direction they are facing relative to the radar beam. Therefore, insects’ flight can cause variation of RCS because of the variation of their aspect angles [15]. Otherwise, cloud, atmosphere and other meteorological conditions can reflect radar clutter. All above factors could affect the extraction accuracy of phase information. Problems such as phase ambiguity and phase jump would occur, so suitable filtering method should be adopted to reduce influence of stochastic phase noise.

### 2.5. Angle Measurement with Multi-Baselines Interferometry

To acquire 3-D coordinate of individual insect, angle measurements of the azimuth and elevation angles are also needed. Based on multi-baselines interferometry theory, range differences between the target and different antennas can be used to take angle measurement. Contrast to the DOA method, which is based on the phase interferometry, the proposed method has an advantage of no phase ambiguity and angle measurement accuracy can be guaranteed with high accuracy range information [13,23].

**Figure 2:**
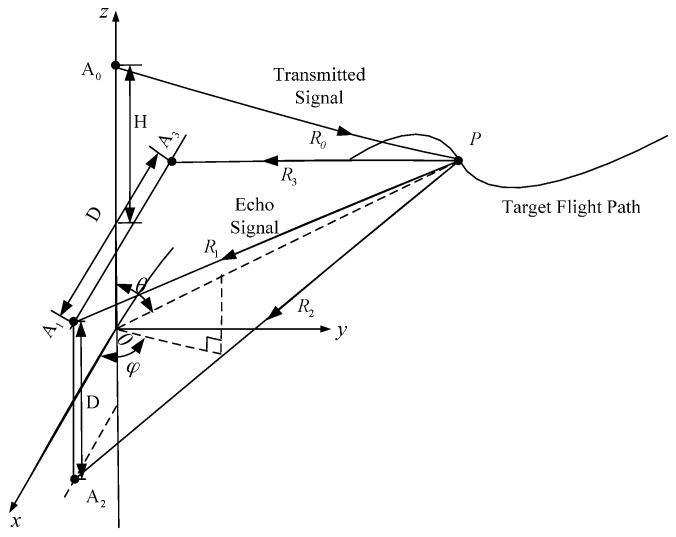
Diagram of angle measurement with multi-baselines interferometry.

Diagram of angle measurement with multi-baselines interferometry is shown in Figure 2. The radar system includes three receiving antennas and one transmitting antenna. The transmitting antenna $A_{0}$ is on the *z*-axis and the three receiving antennas $A_{1}$, $A_{2}$ and $A_{3}$ can form two independent baselines. We adopt antennas $A_{1}$, $A_{2}$ and $A_{1}$, $A_{3}$ to form baselines $A_{1}A_{2}$ and $A_{1}A_{3}$, respectively. Ranges from the target to the four antennas are $R_{0}$, $R_{1}$, $R_{2}$ and $R_{3}$, respectively. Considering that insect migration occurs at heights of up to (and sometimes over) 2 km, the ranges are much larger than the length of the baselines (decimeter level). Therefore, angle measurement can be realized with the equations,

(21) $$\Delta R_{1}=D\cdot \cos\theta ,$$

(22) $$\Delta R_{2}=D\cdot \cos\varphi \cdot \sin\theta ,$$

where $\Delta R_{1}=R_{2}-R_{1}$ and $\Delta R_{2}=R_{3}-R_{1}$ represents the range differences and *D* is the baseline length. Baseline $A_{1}A_{3}$ is parallel to the *x*-axis and $A_{1}A_{2}$ is parallel to the *z*-axis. *H* is the vertical height from transmitting antenna $A_{0}$ to the baseline $A_{1}A_{3}$. θ is the elevation angle between the echo direction and *z*-axis, and φ is the azimuth angle between the projection direction of the echo signal in the *x-y* plane and *x*-axis.

Utilize Equations (21) and (22) to get θ and φ,

(23) $$\left\{ \begin{array}{l} \theta =\arccos\left(\Delta R_{1}/D\right)\\ \varphi =\arccos\left(\Delta R_{2}/\left(D\sin\theta \right)\right) \end{array}\right.$$

The central point of the baseline $A_{1}A_{3}$ is the coordinate origin *O*. Once $R_{1}$ and $R_{3}$ are acquired accurately, range *R* from the target *P* to the origin *O* can be obtained, so the target’s coordinate (R,θ,φ) in the polar coordinate system can be determined. The target’s coordinate $\left(x_{T},y_{T},z_{T}\right)$ in the rectangular coordinate system can be obtained with

(24) $$\{\begin{array}{l} x_{T}=R\cdot \sin\theta \cdot \cos\varphi \\ y_{T}=R\cdot \sin\theta \cdot \sin\varphi \\ z_{T}=R\cdot \cos\theta \end{array}.$$

## 3. Simulated and Experimental Validation

### 3.1. Simulated Validation

#### 3.1.1. Simulation Results

To validate the feasibility of the proposed method to acquire 3-D flight trajectory of individual insect, simulated datasets based on MATLAB are processed. The simulation scenario is shown in Figure 2. The target *P* makes a curve movement in the 3-D space. The height difference *H* is 1.1 m and the length of the baseline *D* is 0.4 m.

Table 1 shows the basic parameters of the S-band radar system. The time duration of each frame of stepped-frequency signal is $T_{F}=NT_{r}=102.4\;\mu s$, the range resolution is ΔR=0.469 m and the wavelength is λ=0.0909 m.

**Figure 3:**
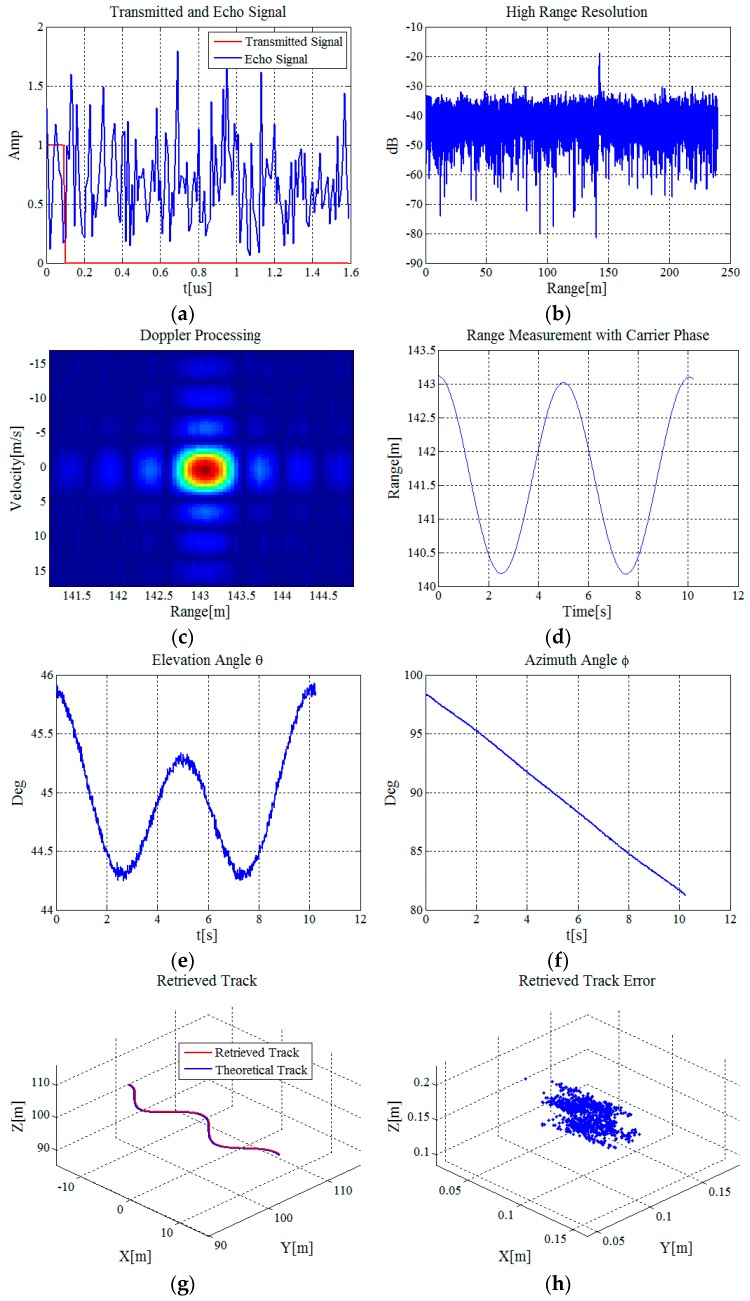
Simulation results: (**a**) Amplitude curves of the transmitted and echo signals; (**b**) dB image of the high resolution synthesizing result; (**c**) Doppler coherent processing result; (**d**) high accuracy range measurement result with carrier phase; (**e**) retrieved result of the elevation angle; (**f**) retrieved result of the azimuth angle; (**g**) retrieved result of the target’s 3-D track; and (**h**) retrieved error of the target’s 3-D track.

Under the condition that the target’s radial velocity in the simulation is no more than v=3 m/s, the value range of M is M≤217 according to Equation (11). M=100 is selected. Projection components of the flight track of the target *P* along the three axes are x(t)=−15+3t, y(t)=100+2cos(2π/5t) and z(t)=100+sin(πt/10), respectively. The time range is 0<t<10 s. Simulation results are shown in Figure 3.

Figure 3a shows that in an ideal case, the amplitude of the transmitted signal is a rectangular wave. However, under the condition of strong stochastic noise, it is hard to determine the target just by the amplitude of the echo signal. As shown in Figure 3b, with high resolution synthesizing, the SNR can be increased obviously to about 15 dB and the target can be detected with the max amplitude. Figure 3c shows the Doppler coherent processing result. The detection performance of the target can be improved further in the 2-D joint domain of range domain and velocity domain. The curve in Figure 3d shows the measurement result of range *R*_1_ between the target *P* and the receiving antenna *A*_1_. To evaluate the measurement accuracy, we analyze the differences between the measured and theoretical ranges. The mean value and standard deviation of the differences are 0.083 mm and 0.045 mm, respectively. The range measurement accuracy is superior to millimeter level. The curve in Figure 3e shows the retrieved result of the elevation angle and it is noisy because of the range measurement error. The curve in Figure 3f shows the retrieved result of the azimuth angles. Since the target *P* moves along the positive direction of the *x*-axis, its azimuth angle decreases with time. The red and blue lines in Figure 3g show the retrieved and theoretical results of the target’s 3-D track, respectively. The measurement error of the target’s 3-D coordinate is shown in Figure 3h. The mean values of the measurement error of the target’s *x*-, *y*-, and *z*- coordinates are −0.757 cm, −4.152 cm and 4.151 cm, with standard deviations 2.120 cm, 2.094 cm and 2.049 cm, respectively. Therefore, the measurement precision can reach centimeter level.

From the simulation results above, it can be noted that the 3-D flight track of a target under the condition of strong stochastic noise can be acquired accurately with the proposed method and the measurement accuracy of the target’s 3-D coordinates can reach centimeter level.

#### 3.1.2. Methods Comparison

To better prove the effectiveness of the proposed method, detailed comparisons are made with other methods.

##### Range Measurement with Time Delay

The most important step of the proposed method is the high accuracy range measurement with carrier phase. Although the range information can be acquired with the time delay of the echo signal according to the location of the max amplitude of the Doppler coherent processing results, the accuracy of range measurement is not high enough for the retrieval of angle information and can affect the accurate acquisition of 3-D trajectory information.

**Figure 4:**
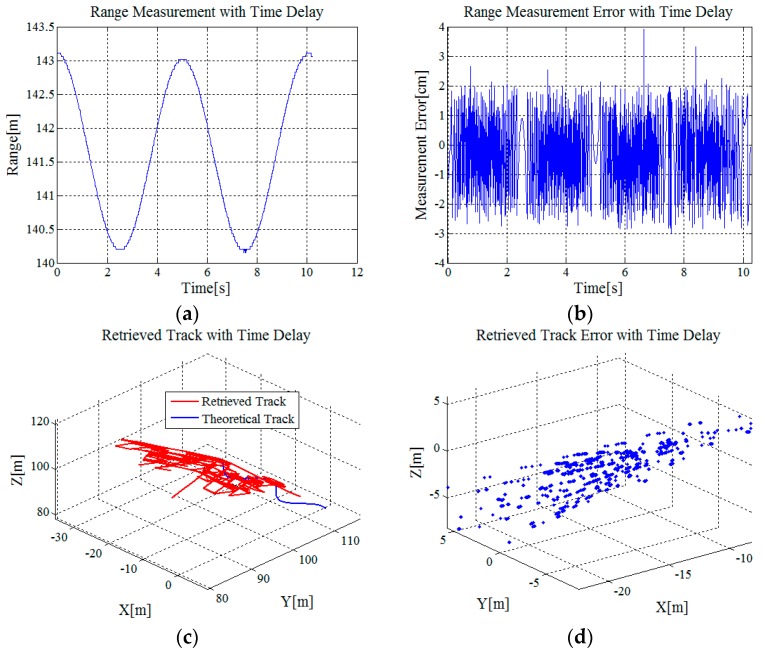
Measurement results: (**a**) Range measurement result with the time delay; (**b**) range measurement error; (**c**) retrieved result of the target’s 3-D track with range information derived from the time delay; and (**d**) retrieved error of the target’s 3-D track.

Figure 4 shows the range measurement result and retrieved result of the target’s 3-D track with range information derived from the time delay of the echo signal. Figure 4a shows the range measurement result and Figure 4b shows the range measurement error. The range resolution of the S-band radar system is ΔR=0.469 m. Although the Doppler processing results are ten-times oversampled in the range direction, the largest measurement deviation is about ΔR/2/10=2.345 cm. By contrast, the range measurement accuracy with carrier phase is superior to millimeter level. Figure 4c shows the retrieved results of the target’s track and Figure 4d shows the corresponding retrieved error. Via statistics, the mean values of the retrieved error of the target’s *x*-, *y*-, and *z*-coordinates are −14.46 m, −1.89 m and 0.66 m, with standard deviations 3.45 m, 3.40 m and 2.36 m, respectively. The measurement accuracy is worse than meter level. It can be noted that accurate range information is quite critical for the acquisition of 3-D trajectory information.

##### TSOA Method

For the TSOA method, the time summations of arrival of an incoming signal by three or more radars are processed to determine the location of a target. In 3-D space, the range summation of the target to two radars defines one ellipsoid. A minimum of three ellipsoids are required to acquire the intersection, which defines the target’s location [14]. Utilizing the S-band radar system shown in Figure 2, the range summation can be defined as the summation of the range from the target to the transmitting antenna and that from the target to one receiving antenna. Therefore, the transmitting antenna $A_{0}$ and three receiving antennas $A_{1}$, $A_{2}$ and $A_{3}$ can be combined together to realize the TSOA algorithm. It can be expressed as,

(25) $$\left\{ \begin{array}{l} \sqrt{{\left(x_{0}-x_{T}\right)}^{2}+{\left(y_{0}-y_{T}\right)}^{2}+{\left(z_{0}-z_{T}\right)}^{2}}+\sqrt{{\left(x_{1}-x_{T}\right)}^{2}+{\left(y_{1}-y_{T}\right)}^{2}+{\left(z_{1}-z_{T}\right)}^{2}}=R_{S1}\\ \sqrt{{\left(x_{0}-x_{T}\right)}^{2}+{\left(y_{0}-y_{T}\right)}^{2}+{\left(z_{0}-z_{T}\right)}^{2}}+\sqrt{{\left(x_{2}-x_{T}\right)}^{2}+{\left(y_{2}-y_{T}\right)}^{2}+{\left(z_{2}-z_{T}\right)}^{2}}=R_{S2}\\ \sqrt{{\left(x_{0}-x_{T}\right)}^{2}+{\left(y_{0}-y_{T}\right)}^{2}+{\left(z_{0}-z_{T}\right)}^{2}}+\sqrt{{\left(x_{3}-x_{T}\right)}^{2}+{\left(y_{3}-y_{T}\right)}^{2}+{\left(z_{3}-z_{T}\right)}^{2}}=R_{S3} \end{array}\right.,$$

where $\left(x_{0},y_{0},z_{0}\right)$, $\left(x_{1},y_{1},z_{1}\right)$, $\left(x_{2},y_{2},z_{2}\right)$ and $\left(x_{3},y_{3},z_{3}\right)$ are the 3-D coordinates of the transmitting antenna $A_{0}$ and receiving antennas $A_{1}$, $A_{2}$, and $A_{3}$, respectively. $\left(x_{T},y_{T},z_{T}\right)$ is the target’s coordinate., and $R_{S1}$, $R_{S2}$, and $R_{S3}$ are the corresponding range summations.

**Figure 5:**
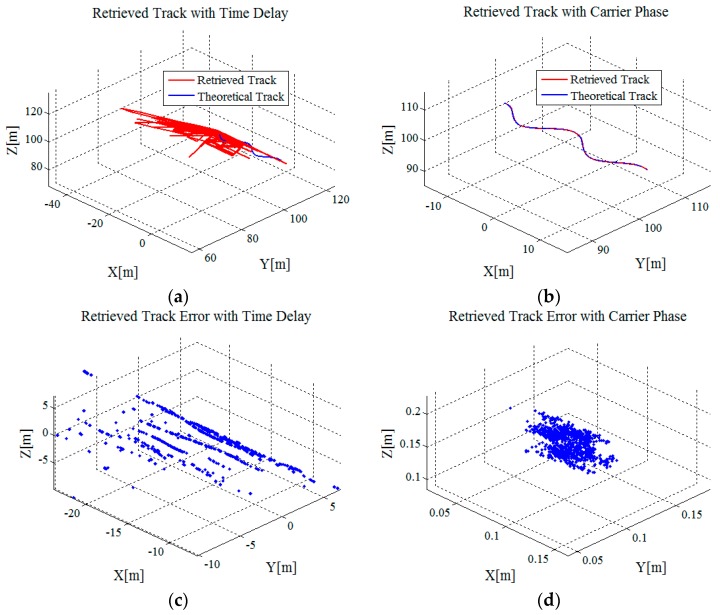
Measurement results: (**a**) Retrieved result of the target’s 3-D track with range information derived from the time delay; (**b**) retrieved result of the target’s 3-D track with range information derived from the carrier phase; (**c**) retrieved error of the target’s 3-D track with range information derived from the time delay; and (**d**) retrieved error of the target’s 3-D track with range information derived from the carrier phase.

To better evaluate the TSOA method, range information derived from the time delay and that derived from the carrier phase are both utilized to retrieve the target’s 3-D track based on Equation (25). Simulation results are shown as Figure 5. Obviously, accurate range information benefits the retrieval of the target’s 3-D track. With range information derived from the carrier phase, the mean values of the retrieved error of the target’s *x*-, *y*-, and *z*-coordinates are −0.75 cm, 4.15 cm and 4.15 cm, with standard deviations 2.12 cm, 2.09 cm and 2.05 cm, respectively. Compared with the simulation results with the proposed method shown in Section 3.1.1, the statistical results of the measurement errors show that the measurement accuracy of our method is almost the same as that of the TSOA method.

The TSOA method utilizes accurate calculating formulas to realize target localization. To solve the non-linear equations as shown in Equation (25), an iteration method is commonly used. However, this is computationally time-consuming and an initial iteration value close to the true solution is required, which might be difficult to select in practice. These disadvantages mean the TSOA method cannot be used to acquire the 3-D flight trajectory of individual insect in real-time.

##### DOA Method

For the DOA method, the direction of arrival of an incoming signal is determined with a multi-baselines interferometer [13]. The azimuth and elevation angles of a target are retrieved by measuring the phase differences between different antennas. Two independent baselines can be formed with the S-band radar system shown in Figure 2. The phase differences related with the two baselines can be expressed as follows:

(26) $$\left\{ \begin{array}{l} \Psi_{12}=\left(2\pi D/\lambda \right)\cos\theta \\ \Psi_{13}=\left(2\pi D/\lambda \right)\sin\theta \cos\varphi \end{array}\right.,$$

where $\Psi_{12}$ is the phase difference of echo signals received by the receiving antennas $A_{1}$ and $A_{2}$, and $\Psi_{13}$ is the phase difference by the receiving antennas $A_{1}$ and $A_{3}$. θ and φ are the spherical coordinates specifying the direction of arrival of the echo signal. If $\Psi_{12}$ and $\Psi_{13}$ are known, the direction of arrival of the echo signal can be computed.

However, when the baseline of the interferometer is larger than λ/2, the possible range of phase differences can exceed 2π. This leads to an ambiguity in determining the direction of arrival of the echo signal. The baseline of the S-band radar system is D=0.4 m, and the wavelength is λ=0.0909 m. The length of the two baselines D almost equals 4λ, and therefore the problem of phase ambiguity cannot be avoided when taking DOA measurement with the S-band system.

**Figure 6:**
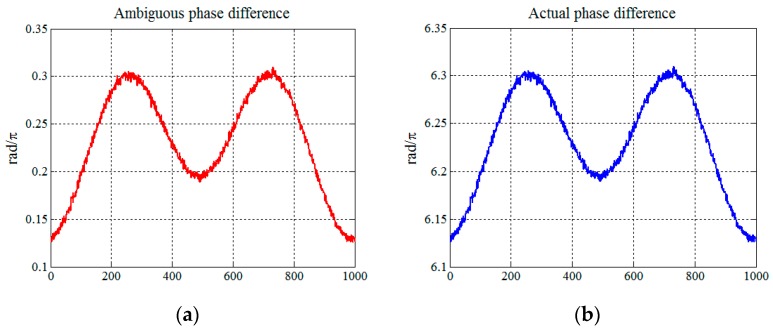
Measurement results: (**a**) Ambiguous phase difference of the echo signals from the receiving antennas $A_{1}$ and $A_{2}$; and (**b**) actual phase difference.

Figure 6 shows the simulation results of the phase differences of echo signals from the receiving antennas $A_{1}$ and $A_{2}$. It can be noted that the ambiguous and actual phase differences are different, and measurement results indicate a difference of 6π. The phase ambiguity has to be resolved before taking the angle measurement. Unfortunately, the S-band radar system lacks extra antennas to help resolve the phase ambiguity. The DOA method has disadvantage of phase ambiguity and should only be applied to some specific radar systems.

From the comparison results above, it can be noted that the modification to take range measurement with the carrier phase rather than the time delay benefits the acquisition of the target’s 3-D track significantly. The retrieval accuracy of the target’s 3-D coordinates can be improved from meter level to centimeter level. As for the TSOA method, it is built upon accurate non-linear equations. An iteration method is commonly adopted to solve the non-linear equations. The process is much more computationally time-consuming than the proposed method, and an initial iteration value close to the true solution is required, which might be difficult to select in practice. These disadvantages mean the TSOA method cannot satisfy the need of acquiring 3-D flight trajectory of individual insect in real-time. Moreover, the measurement accuracy of the TSOA method is almost the same with the proposed method. As for the DOA method, it has an obvious disadvantage of phase ambiguity and should be applied to some specific radar systems. In conclusion, simulation results prove that the proposed method can acquire 3-D flight trajectory of individual insect with high accuracy. Besides, it is easy to be realized without problems such as time consuming and phase ambiguity.

### 3.2. Experimental Validation

**Figure 7:**
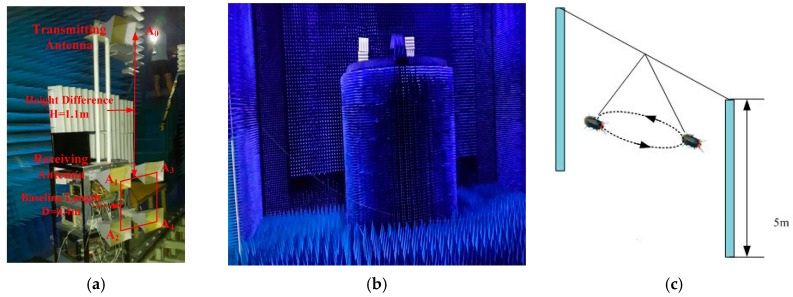
Experimental scene: (**a**) Photo of the S-band radar system; (**b**) photo of the experimental scene in the anechoic chamber; and (**c**) diagram of insect flying.

To validate the feasibility of the proposed method to acquire the 3-D flight trajectory of individual insect in practice, experimental datasets acquired in the anechoic chamber are processed. Experimental scene is shown in Figure 7. Figure 7a shows the photo of the S-band radar system including one transmitting antenna and four receiving antennas, three of which are utilized. System parameters and the coordinate system keep the same with the simulated scene. The radar parameters are shown in Table 1. Figure 7b shows the photo of the anechoic chamber. Since we cannot let the insects fly free in the anechoic chamber, we put two plastic poles about 5 m high at both sides of the anechoic chamber. A cotton thread is tied to the top end of the both poles and an insect is tied to another cotton thread about 1 m long in the middle of the first thread. The insect can still fly, but under the constraint of the second thread, it flies in circles in the experiments. In the experiments, the insects fly at a height of about 3 m to 4 m above the ground and the radial range to the radar varies at the range of 10 m to 15 m. Figure 7c is the diagram of a flying insect. The radar system is put on a high platform and the insects fly below relative to the radar, so their *z*-coordinate is negative.

Table 2 shows the insects’ information in the anechoic chamber experiments, including insects’ names, photos, body sizes and the environment information. We select four typical migratory insects to take experiments. This paper takes a greenish brown hawk as an example to present the experimental results and validate the feasibility of the proposed method.

**Figure 8:**
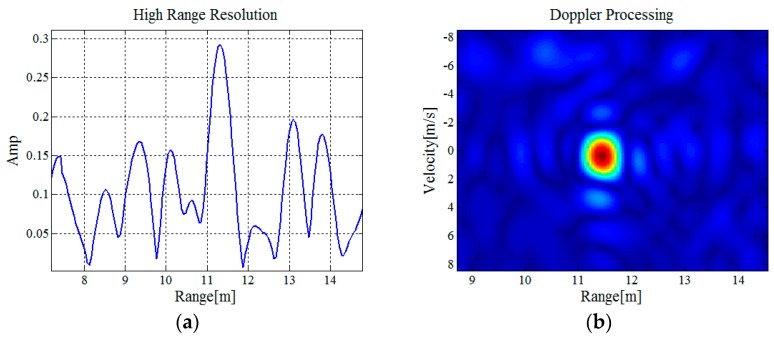

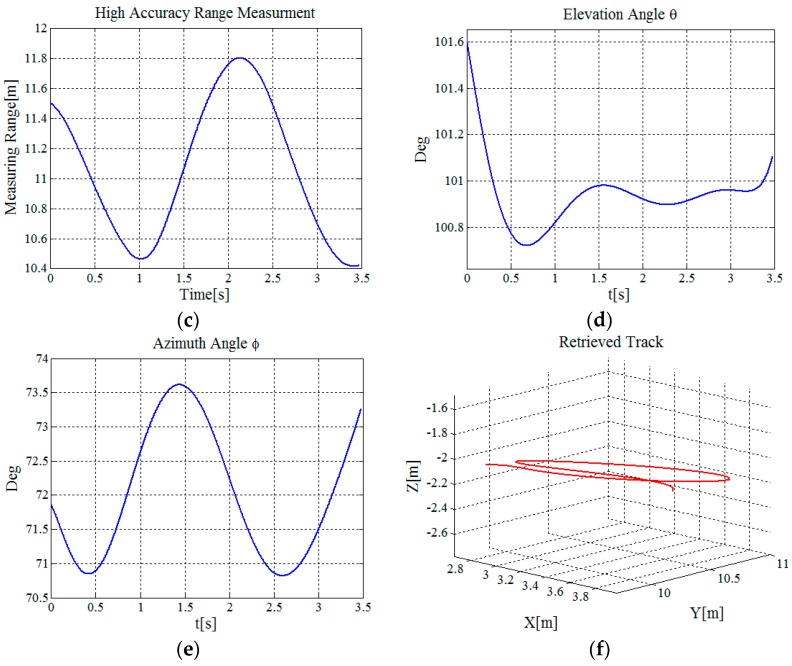
Experimental results: (**a**) Amplitude curve of the high resolution synthesizing result; (**b**) Doppler coherent processing result; (**c**) high accuracy range measurement result with carrier phase; (**d**) retrieved result of the elevation angle; (**e**) retrieved result of the azimuth angle; and (**f**) retrieved result of the insect’s flight track.

Experimental results are shown in Figure 8. Figure 8a shows that contrast to wavelength (9.09 cm) of the S-band radar system, the body length of the insect is shorter than half-wavelength, and echo amplitude of the insects is very small, so it is difficult to detect the insect effectively just by the amplitude of the high resolution synthesizing result. As shown in Figure 8b, with Doppler coherent processing, the SNR is increased obviously and the insect can be detected effectively. Detection performance of the insect as a weak radar target can be improved in the 2-D joint domain of range domain and velocity domain. The curve in Figure 8c shows the measurement result of range $R_{1}$ between the insect and the receiving antenna $A_{1}$. With a time-domain filtering, range measurement accuracy is further improved. The curve in Figure 8d shows the retrieved result of the elevation angle. Under the constraint of a thread, the insect’s flight height changed little. Therefore, the variation of its elevation angle is small. The curve in Figure 8e shows the retrieved result of the azimuth angle. The curve in Figure 8f shows the retrieved result of the insect’s flight trajectory. Considering that the insect flew in circles, the result corresponds to the practical situation. In addition, 3-D coordinates variation of the retrieved result fit well with the actual situation.

## 4. Conclusions

This paper proposes a novel method to acquire the 3-D flight trajectory of individual insect. The most important step is the high accuracy range measurement with carrier phase. Compared with traditional range measurement method with the time delay of the echo signal, the modification to take range measurement with the carrier phase benefits the acquisition of a target’s 3-D track significantly. The retrieval accuracy of a simulated target’s 3-D coordinates can be improved from meter level to centimeter level. To prove the effectiveness of the proposed method, comparison results with other localization methods such as the TSOA method and the DOA method prove that the proposed method is easy to be realized without problems such as time consuming and phase ambiguity. To prove the feasibility of the proposed method, utilizing a high resolution S-band radar system, experimental datasets acquired in the anechoic chamber are processed. Experimental results prove that the Doppler coherent processing can improve SNR significantly and benefit the effective detection of insects. In addition, the retrieved trajectory results show that insects’ flight behaviors and 3-D coordinates’ variation match well with actual situations.

This proposed method provides a novel way to monitor 3-D flight trajectory of the aerial free-fly insects. Forthcoming experimental analysis of 3-D flight trajectory of the aerial free-fly migrating insects will give a better verification of the feasibility of the proposed measurement method, benefiting the research of insect migration behaviors and the development of migratory entomology.

## Figures and Tables

**Table 1:** Parameters of the S-band radar system.

Parameters	Symbol	Value
Number of subpulses	N	64
Subpulse repetition interval	$T_{r}$	1.6 μs
Subpulse duration	$T_{p}$	0.1 μs
Frequency step size	Δf	5 MHz
Carrier frequency	$f_{0}$	3.3 GHz
Sampling frequency	$f_{s}$	100 MHz

**Table 2:** Insects’ information in the anechoic chamber experiments.

Name	Photo	Body Size (mm)		Measurement Environment	
		Body Length	Wing Width	Temperature (°C)	Humidity (%)
Sweet Potato Hornworm	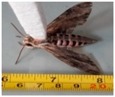	42	61	32	88
Greenish Brown Hawk	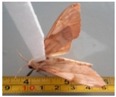	41	77	31	64
Huai Noctuid	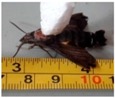	30	30	32	58
Black Cutworm	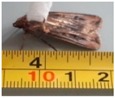	24	13	32	59

## Abbreviations

The following abbreviations are used in this manuscript:

VLR	Vertical-looking radar
DOA	Direction of arrival
TSOA	Time summation of arrival
IDFT	Inverse Discrete Fourier Transform
SNR	Signal-to-Noise ratio
RCS	Radar cross section
DFT	Discrete Fourier Transform
